# Feasibility of Parylene C for encapsulating piezoelectric actuators in active medical implants

**DOI:** 10.3389/fmedt.2023.1211423

**Published:** 2023-11-17

**Authors:** Alina Kohler, Felix Blendinger, Sonja Müller, Ulrich Mescheder, Volker Bucher

**Affiliations:** ^1^ Faculty Mechanical and Medical Engineering (MME), Institute for Microsystems Technology (iMST), Furtwangen University, Furtwangen, Germany; ^2^ Laboratory for Biomedical Microtechnology, Department of Microsystems Engineering-IMTEK, University of Freiburg, Freiburg, Germany; ^3^ BrainLinks-BrainTools Center, University of Freiburg, Freiburg, Germany; ^4^ Department of Microsystems Engineering IMTEK, University of Freiburg, Freiburg, Germany

**Keywords:** Parylene C, encapsulation, piezoelectric actuator, stretchability, active implant, polymer

## Abstract

Parylene C is well-known as an encapsulation material for medical implants. Within the approach of miniaturization and automatization of a bone distractor, piezoelectric actuators were encapsulated with Parylene C. The stretchability of the polymer was investigated with respect to the encapsulation functionality of piezoelectric chips. We determined a linear yield strain of 1% of approximately 12-µm-thick Parylene C foil. Parylene C encapsulation withstands the mechanical stress of a minimum of 5×10^5^ duty cycles by continuous actuation. The experiments demonstrate that elongation of the encapsulation on piezoelectric actuators and thus the elongation of Parylene C up to 0.8 mm are feasible.

## 1. Introduction

In modern medical technology, active implants are state-of-the-art. Examples of well-established implants are cochlear implants or pacemakers. The European Medical Device Regulation (MDR) defines an active implantable medical device (AIMD) as an implant designed to be inserted, in whole or in part, into the human body by a surgical or medical procedure or into a natural body orifice by a medical procedure and intended to remain there after the procedure. Its operation depends on a source of energy other than energy harvested for this purpose by the human body or by gravity, and which acts changing the density or converting this energy. This type of implant requires a high degree of miniaturization for patient compliance and automation for optimal therapeutic effect.

Within this innovative development of AIMDs lies the further development of bone distractors. Specifically, distractor osteogenesis finds application for treating mandibular hypoplasia, a dentofacial deformity requiring a combination of orthodontic and surgical treatment (1). Distraction osteogenesis is an established methodology where new bone tissue is naturally generated between two parts of a fractured bone based on the tension–stress effect proceeding three phases: a latency phase after surgery, the distraction phase with the actual separation of two bone ends, and the consolidation phase forming the new bone tissue (2–4).

During the distraction phase, distractors are manually activated with an adjusting screw through a body opening using a distraction rod. These adjustments must be performed daily for up to 6 months, depending on the treatment. Several complications are associated with mandibular distractor osteogenesis, including the permanent risk of infection due to a constantly existing body orifice, inappropriate distraction vector or inaccurate adjustment leading to pain for the patient or unsuccessful treatment, and interference in the patient's daily life through constant monitoring and adjustment of the distraction process (5–7).

Automation and miniaturization of distractors is a potential optimization to reduce the abovementioned complications to enhance the affected patient's quality of life. Especially within the application of mandibular distraction osteogenesis, the optimization is of utmost importance since the affected individuals suffer not only from often underestimated esthetic issues but also from functional reasons due to craniofacial microsomia.

For example, the principle of a linear inchworm piezoelectric motor can be exploited for this purpose, which is small enough to implant (8). Piezoelectric actuators are used for clamping and changing the position of a shaft, thus keeping two parts of the implant at a defined distance and moving them in a controlled manner. Driving the piezoelectric actuators requires voltages of typically up to several hundred volts (9). This makes it necessary to provide suitable insulation especially when used in medical implants.

Parylene C is a well-known encapsulation polymer for electronic circuits under harsh environments. Due to its advantageous properties, such as chemical robustness, biocompatibility, and sterilizability, it can also be used to encapsulate biomedical implants (10). Deposited by a chemical vapor deposition (CVD) process known as the Gorham process (11), di-*p*-xylylene as a precursor is used to pyrolyze its dimers, generating reactive monomers to sublimate on the surface by polymerization within the gas phase. The resulting homogeneous Parylene C coating serves with its dielectric strength of up to 343 V/µm (12, 13) as an excellent insulating material.

The long-term encapsulation properties have already been tested thoroughly on rigid and flexible implant materials (14–16). Further, Golda-Cepa et al. (17) gave a comprehensive overview of Parylene C for biomedical applications and presented mechanical testing and bending of Parylene C and compound material coatings of different materials (18–20). However, the properties of stretchable materials and devices, specifically of piezoelectric actuators, have not yet been investigated by cyclic stretching.

This work evaluates the feasibility of Parylene C as an encapsulation for piezoelectric actuators in medical implants. We have used commercially available piezoelectric actuators to examine the encapsulation properties through dielectric breakdown tests after a sequential number of actuation cycles of the actuators. Furthermore, the mechanical properties of Parylene C were analyzed through tensile testing of Parylene C foils.

## 2. Materials and methods

### 2.1. Parylene C deposition

Parylene C was deposited using a Labcoater 300 (Plasma-Parylene Systems GmbH, D-Rosenheim). Silane A-174 (Sigma-Aldrich) is used as an adhesion promoter after an O_2_-plasma pretreatment (300 W, 5 min). The pressure during deposition was 2.0–4.0 Pa.

A coating thickness of 11.9 µm was deposited. The thickness was determined on a Si-wafer piece with a UV-Vis Reflectometer (Nanocalc-XR, Oceanoptics). Theoretically, Parylene C is considered pinhole-free starting from 1 µm (10), calculating with a safety factor by a minimum of 10 for including possible defects in layer homogeneity due to the manufacturing process (21).

### 2.2. Mechanical properties of Parylene C

Parylene foils were prepared by coating a Si-wafer with a Parylene C layer. The steps of plasma pretreatment and the application of an adhesion promoter were omitted. Prior to deposition, a release layer was applied to the wafer by rinsing it with dishwashing liquid diluted in deionized water. The coating thickness measured on an untreated wafer piece was 11.9 µm.

Foils of 80.0 × 10.0 mm^2^ were cut using a scalpel. Tensile testing was performed with a 50-N load cell (FMT310, Alluris D-Freiburg) mounted on a universal testing machine (Alluris, D-Freiburg). The Parylene foil was fixed by home-built clamps in the testing machine. The distance between the clamps prior to tensile testing was set to 50.0 mm. The displacement speed was set to 1.0 mm/min.

### 2.3. Encapsulation of piezoelectric chips

**Figure 1:**
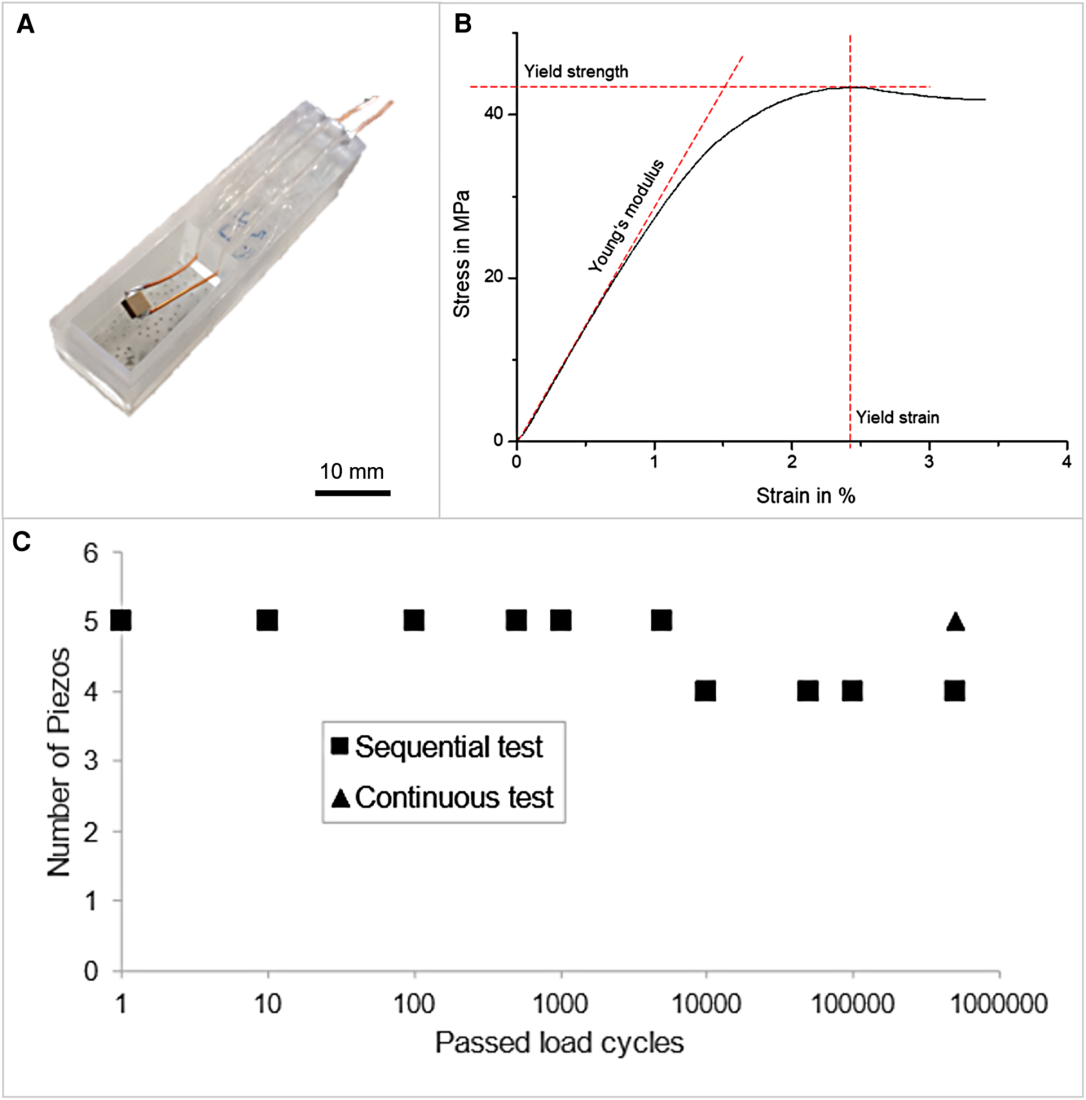
(**A**) Piezoelectric chip contacted with enameled copper wires and placed inside a 3D printed holder. (**B**) Representative stress/strain diagram of Parylene C. (**C**) Examination of the long-term performance of Parylene C encapsulation of piezoelectric actuators obtained by pulsing a specified amount of load cycles and performing continuous and subsequent measurement of the breakdown strength, respectively.

Piezoelectric chips with a height of 2.0 mm and a displacement of 2.0 µm (PA2JEW, Thorlabs) were contacted with enameled copper wires (0.5 mm diameter). Preliminary tests were done using commercially available piezoelectric chips with preattached silicone-insulated wires. The preattached wires were replaced by enameled copper wires for encapsulation optimization. The assembly was placed in a 3D printed holder for protection and easier handling (Figure 1A). Parylene C encapsulation of the piezoelectric chips was performed using the protocol described in Section 2.1.

### 2.4. Duty cycle test process

The longtime stability was investigated for 10 Parylene C-coated piezoelectric actuators. Five of the coated actuators were subjected to a sequential duty cycle test process to determine the longtime stability. In addition, five coated actuators were tested by one continuous sequence of 5×10^5^ duty cycles. Preliminary to each duty cycle, the dielectric strength was tested by the protocol mentioned in Section 2.5.

To deform the actuators and stress the coating, a square wave with a frequency of 1 Hz was generated by a function generator (Voltcraft 7202). The low frequency was chosen to avoid resonance effects and to ensure a maximum displacement of the actuator. In addition, 1 Hz is close to the frequency expected to be used in the medical implant. The wave signal was amplified using a piezo controller (MDT693B, Thorlabs) such that the actuators were powered with a voltage between 0 V for the low half-wave and 75 V for the high half-wave. After each sequence of applied duty cycles, the dielectric strength was checked for malfunction of the Parylene C coating. The number of sequential duty cycles ranged between 1 and 5×10^5^.

### 2.5. Measurement of the breakdown strength

The breakdown strength of the coated piezo chips was measured with an ST920IC high-voltage tester (Sourcetronic GmbH, Germany) before and after each duty cycle test process. An alternating voltage of 50 Hz was increased linearly to 75 V_rms_ within 30 s, held for 60 s, and returned to 0 V within 30 s. The test was considered passed if a current of 10 µA was not exceeded. This threshold was set based on the setup's internal leakage current.

### 2.6. Failure analysis

Localization of coating failures after a failed breakdown test was performed by electrodeposition of copper. The sample was placed in a copper acetate solution, and a bias voltage of up to 5 V was applied. As soon as a current of 50 µA was reached, the electroplating process was stopped.

## 3. Results

### 3.1. Mechanical properties of Parylene C

The stress/strain curve obtained by tensile testing of five foils is shown in Figure 1B. The stress increased with applied strain. Until approximately 1% strain, a linear increase was observed. A maximum stress was reached with a further increase of strain, resulting in a yield strength of 42.5 ± 1.1 MPa. The stress decreased once the yield strain of 2.58 ± 0.17% was exceeded. For the tested samples, Young's modulus of 2.81 ± 0.06 GPa was obtained.

### 3.2. Long-term performance of Parylene C applied to piezoelectric actuators

The performance of Parylene C encapsulation on piezoelectric actuators was evaluated by applying a specified number of pulses to the actuators and then investigating the insulation properties of the encapsulation by measuring the dielectric strength at 75 V_RMS_, see Figure 1C. This process was repeated until an encapsulation failure was measured.

As shown in Figure 1C, the encapsulation properties of five actuators were evaluated in a sequential test with several intermediate steps until a total number of 5×10^5^ cycles were reached. For one actuator, the encapsulation failed after 5×10^3^ cycles. In a second test, five actuators were loaded with continuous 5×10^5^ cycles, and no actuator failed.

### 3.3. Challenges and stability of encapsulation—preattached silicone-insulated wires

**Figure 2:**
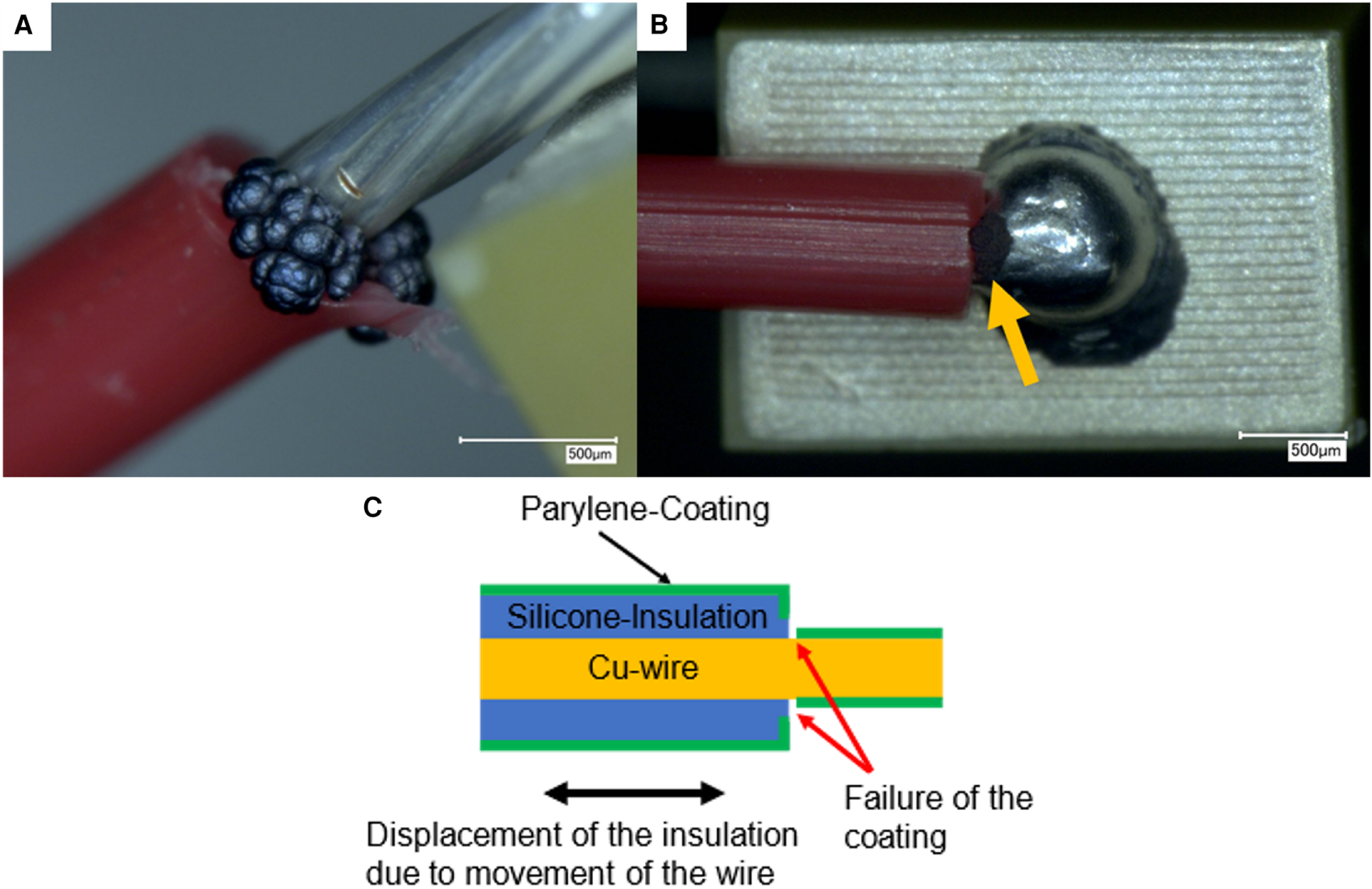
(**A,B**) Optical micrographs of the failure of the Parylene C coating at the insulation of the contact lead from two different piezo samples. The coating failure is visible after electroplating with copper through a gray deposit. (**C**) Suggested failure mechanism of the Parylene C coating on copper wires insulated by a silicone sheath due to movement of the insulation.

Previous dielectric strength measurement revealed that the encapsulation of the piezoelectric chips with the preassembled insulated silicone wires was defective even before the chips were pulsed for the first time. Defect analysis through copper electrodeposition revealed defects in the area where the wire was stripped of its insulation (Figures 2A,B).

For these experiments using piezoelectric chips connected with enameled copper wires, no other failure of the encapsulation on the piezoelectric actuators at the critical connection point between the wire and chip was observed before actuation.

## 4. Discussion

Determining the mechanical properties of the Parylene C foils provided information about the produced layers with a thickness of 11.9 µm. The Youngs modulus was 2.81 GPa, which is within the values in the literature of Hassler et al. (16) and von Metzen and Stieglitz (22). The yield strength of 42.5 MPa was confirmed by Hassler et al. Only the yield strain in the experiments performed here was 2.58%, which is close to half of the value (4.0%) determined by Metzen et al. and nearly a third of the minimum yield strain (7.5%) determined by Hassler et al.

Based on the literature data, a correlation between the thickness and an increasing Young's modulus could be assumed, but this assumption could not be confirmed with the varied yield strength, yield strain, and deposition pressure values. Hence, the coating conditions are of high relevance, causing discrepancies in the resulting mechanical properties. The deposition pressure influences the crystallinity of the polymer by the formation of more polymerization centers with higher deposition pressure, thus forming more and shorter polymer chains, which leads to more amorphous Parylene C and increases the probability of inclusions and voids (16, 23). In addition, possible contamination in the precursor could lead to inhomogeneity in the molecular structure of the layer or a higher inclusion of the processing gas (O_2_ or N) during deposition, more precisely within the polymerization phase on the surface (11).

However, the linear range of the resulting strain is within 1%. Important for the investigations performed here is that the maximum displacement of a piezo used in the experiments, is 2 µm. This value within the linear yield strain of 1% is equivalent to 0.8 mm; thus, the Parylene C coating is sufficiently adequate for this application of a piezoelectric actuator.

Duty cycle test procedures were used to test the long-term behavior of the Parylene C coating on the piezoelectric actuators. The encapsulation layer thickness on the piezoelectric actuators of 11.9 µm was equivalent to the preliminary tests of the Parylene C foils. The pulsing tests correspond to real conditions of the application for the time a distractor is being implanted. This can be up to 6 months, which corresponds to 5×10^5^ pulses. Both the sequential and continuous duty cycles were passed by nine piezo chips. Only one actuator passed the sequential run of up to 5×10^3^ duty cycles. The outlier could have been caused by handling or possible weak points at the soldered junction with the copper wires. A failure analysis with copper electrodeposition showed the failure of the layer on named areas. Despite this single sample, it was shown that Parylene C has useful mechanical properties as an encapsulation layer for expandable electrical components up to an elongation of 0.8 mm.

The measurements were explicitly limited to the voltage of 75 V because the Parylene C encapsulation of the actuators plays a role in additional insulation protection of a planned miniaturized and automated distractor prototype, thereby providing further not necessarily needed insulation. And yet, this additional insulation layer oblige warranty for the patient's safety. The piezoelectric stacks are supposed to be mounted within mechanically sliding rails aimed to be encapsulated by a flexible biocompatible bellows (24). Hence, the measurement does not meet the requirements of standard DIN EN ISO 60601-2-2, ensuring the safety of patients and users by a breakdown voltage of more than 1.0 kV of the electrical insulation layer. However, this is secondary at this stage of manufacturing, considering that the Parylene C layer on the actuators is the additional encapsulation safety factor.

In the investigation of the encapsulation of piezo actuators with preattached silicone-insulated copper wires, failures of the coating were found in the region where the insulation was stripped. The coating might have failed in this area because of possible movement of the insulation on the copper wire (Figure 2C) during handling of the piezo samples and movement of the wire. The yield strain of Parylene C does not seem to be sufficient to accommodate the movement of silicone insulation, leading to a shearing of the coating and exposure of the copper wire that was covered by the silicone insulation during the Parylene deposition step.

No such defects were found in the long-term experiments. In these experiments, enameled copper wires were used for contacting the piezo actuators, and thus, no movement of the enamel on the copper wires had to be expected.

## 5. Conclusion and outlook

Within these experiments, it was shown that Parylene C as an encapsulation material of 11.9 µm thickness for piezoelectric chips withstands the mechanical stress of actuation of a minimum of 5×10^5^ duty cycles. Based on additional tensile tests with Parylene C foils and the determined linear strain of 1%, it can be concluded that an elongation of piezoelectric actuators of up to 0.8 mm is possible without failure of the Parylene C layer.

Discrepancies of the tensile test results with Refs. (16) and (22) might be explained by slightly different coating conditions. Therefore, further investigations of the influences of deposition pressure and coating thickness on the mechanical properties must be conducted.

Care must be taken when choosing suitable electrical cable connections, as the Parylene C coating seems susceptible to silicone-insulated wires, leading to defective insulation.

## Data Availability

The original contributions presented in the study are included in the article/Supplementary Material, further inquiries can be directed to the corresponding authors.
